# Predicting Cardiac Arrest in Children with Heart Disease: A Novel Machine Learning Algorithm

**DOI:** 10.3390/jcm12072728

**Published:** 2023-04-06

**Authors:** Priscilla Yu, Michael Skinner, Ivie Esangbedo, Javier J. Lasa, Xilong Li, Sriraam Natarajan, Lakshmi Raman

**Affiliations:** 1 Division of Critical Care, Department of Pediatrics, University of Texas Southwestern Medical Center, Dallas, TX 75235, USA; 2 Department of Computer Science, University of Texas at Dallas, Richardson, TX 75080, USA; 3 Department of Surgery, University of Texas Southwestern Medical Center, Dallas, TX 75235, USA; 4 Section of Cardiac Critical Care, Division of Critical Care Medicine, Department of Pediatrics, University of Washington, Seattle, WA 98195, USA; 5 Division of Cardiology, Department of Pediatrics, University of Texas Southwestern Medical Center, Dallas, TX 75235, USA; 6 Peter O’Donnell Jr. School of Public Health, University of Texas Southwestern Medical Center, Dallas, TX 75235, USA

**Keywords:** machine learning, cardiac arrest, prediction, pediatrics, heart disease

## Abstract

Background: Children with congenital and acquired heart disease are at a higher risk of cardiac arrest compared to those without heart disease. Although the monitoring of cardiopulmonary resuscitation quality and extracorporeal resuscitation technologies have advanced, survival after cardiac arrest in this population has not improved. Cardiac arrest prevention, using predictive algorithms with machine learning, has the potential to reduce cardiac arrest rates. However, few studies have evaluated the use of these algorithms in predicting cardiac arrest in children with heart disease. Methods: We collected demographic, laboratory, and vital sign information from the electronic health records (EHR) of all the patients that were admitted to a single-center pediatric cardiac intensive care unit (CICU), between 2010 and 2019, who had a cardiac arrest during their CICU admission, as well as a comparator group of randomly selected non-cardiac-arrest controls. We compared traditional logistic regression modeling against a novel adaptation of a machine learning algorithm (functional gradient boosting), using time series data to predict the risk of cardiac arrest. Results: A total of 160 unique cardiac arrest events were matched to non-cardiac-arrest time periods. Using 11 different variables (vital signs and laboratory values) from the EHR, our algorithm’s peak performance for the prediction of cardiac arrest was at one hour prior to the cardiac arrest (AUROC of 0.85 [0.79,0.90]), a performance that was similar to our previously published multivariable logistic regression model. Conclusions: Our novel machine learning predictive algorithm, which was developed using retrospective data that were collected from the EHR and predicted cardiac arrest in the children that were admitted to a single-center pediatric cardiac intensive care unit, demonstrated a performance that was similar to that of a traditional logistic regression model. While these results are encouraging, future research, including prospective validations with multicenter data, is warranted prior to the implementation of this algorithm as a real-time clinical decision support tool.

## 1. Introduction

The prevention and management of hospital cardiac arrest (IHCA) in children with congenital and acquired heart disease remains a challenging problem. Among children who are hospitalized, cardiac arrest occurs at a ten-fold higher rate in children with heart disease than those without heart disease [1]. Of the patients who have had an IHCA in a pediatric cardiac intensive care unit (CICU), medical encounters had a survival of 37.7%, while surgical encounters had a survival of 62.5% [2]. Accordingly, the 2018 American Heart Association Scientific Statement on CPR on children with cardiac disease placed emphasis on the prearrest phase and prevention of cardiac arrest, given the poor outcomes once IHCA occurs [3]. In children with critical congenital heart disease (CHD) (defined as lesions requiring surgery or catheter-based interventions in the first year of life), abnormal arterial and venous circulations likely contribute to CPR inefficiencies, but may provide unique signals that may be identifiable, given the current monitoring in subspecialized care units [3]. 

Challenges exist in identifying which patients will suffer from an IHCA. With the available patient data from the electronic heath records (EHR), our group has previously demonstrated a successful prediction model development for IHCA within the pediatric cardiac population, through the use of a multivariable logistic regression model [4]. While the literature using such algorithms to predict IHCAs is growing [5], there are limited studies looking at predicting IHCA in the broader pediatric population [6,7], as well as those with heart disease [8,9,10,11]. Where the majority of studies using machine learning algorithms to predict IHCA involve using continuous (high frequency) physiologic monitoring, one study has shown that routinely collected data from the EHR (i.e., vital signs and laboratory values) can detect adverse events up to 8 h prior to an IHCA in patients with critical CHD (i.e., single ventricle physiology) [9]. We believe that the addition of other key variables, such as demographics and medications, to these predictive analytic algorithms can improve their accuracy and thus prepare clinicians to intervene earlier and possibly prevent an IHCA. Thus, our primary objective is to evaluate if a machine learning algorithm can accurately predict an IHCA in patients that have been admitted to a single-center pediatric CICU, utilizing routinely collected information from the EHR. Furthermore, recognizing that the physiologic and laboratory values are related to the measurements taken at previous time points, we exploited the machinery of statistical relational learning (SRL) [12] to explore whether predictive models, in which patient data are encoded as relational predicates/tables (rather than as vectors), exhibit modelling advantages over standard feature vector based machine learning models.

## 2. Methods

### 2.1. Study Population

The study was conducted in accordance with the Declaration of Helsinki, and approved by the Institutional Review Board of University of Texas Southwestern Medical Center (protocol code STU 2019-0693; date of approval 5/17/19). All the cardiac arrests resulting in CPR that occurred in the pediatric CICU, between November 2010 and January 2019, were captured from a local database of CPR events. For the purpose of this study, we selected index events only. We selected control (non-IHCA) patients at random with the following criteria: (1) The patient had to have been admitted to the CICU between November 2010 and January 2019, (2) did not have a cardiac arrest during the current admission, (3) was not in the cardiac arrest group in prior or future admissions, and (4) was not placed on extracorporeal membrane oxygenation (ECMO) during the first 48 h of their admission. Our initial analysis involved selecting a 1:10 case-to-control ratio. However, after the removal of the control patients who did not meet the inclusion criteria, the final case-to-control ratio was approximately 1:5.

### 2.2. Study Variables

The comprehensive data that were collected from the EHR included vital signs, ventilator settings, laboratory values, urine output, and medications (Supplemental Table S1). All the values were captured, regardless of the frequency of the data input into the EHR. The frequency of the data that were collected in the EHR depended on the type of data and how critically ill the patient was. At a minimum, all CICU patients have hourly vital signs, continuous infusions of medications, and mechanical ventilator settings documented in the EHR, however, this may occur more frequently if they are felt to be more unstable. The frequency of the data collection of all the other features, such as the laboratory values, were variable, with no minimum frequency and a high dependency on the stability of the patient. In place of the individual vasoactive medications, we used the vasoactive inotrope score (VIS). The VIS was defined by the equation: Dopamine dose (mcg/kg/min) + Dobutamine dose (mcg/kg/min) + (100 × Epinephrine dose (mcg/kg/min)) + (10 × Milrinone dose (mcg/kg/min)) + (10,000 × Vasopressin dose (units/kg/min)) + (100 × Norepinephrine dose (mcg/kg/min)). For the patients who had an IHCA, we collected their data from the EHR up to 48 h prior to the arrest event. For the control patients, we collected their data for up to the first 48 h of their CICU admission, since we believe that the first 48 h of admission is when patients have the most risk of instability. Where data were missing, values were imputed from the previous measurement. We have previously published the amount of missing data by feature from this dataset [4]. There was no cutoff on the amount of missing data for an IHCA or control event to be excluded from use in our predictive algorithm. We believe that missing data is representative of how the actual patient care was performed. Clinicians often have to make decisions based on limited data points. For the creation and evaluation of the machine learning predictive model, a five-fold cross validation was performed; thus, for each model, 80% of the patients were used for training and 20% for testing. Since we did not have a separate test set (ideally from a different population), our validation sets in each fold served as test sets. The results were then averaged across these folds.

We collected the demographic data on all the patients. Age, weight, gestational age, illness category (surgical vs. medical), and the type of congenital heart disease, stratified by the ventricular chamber and physiology, were collected from the EHR. Single-ventricle physiology is defined as those patients with mixture of systemic venous and pulmonary venous return, in which their cardiac output is partitioned into the parallel pulmonary and systemic blood flow [13]. This definition would include patients who underwent single-ventricle palliative surgical procedures, as well as patients that were palliated initially with a systemic pulmonary artery shunt before a two-ventricle repair (such as Tetralogy of Fallot). The information related to the cardiac surgeries, i.e., the date of the surgery, the STS-European Association for Cardiothoracic Surgery (STS-EACTS) mortality category (STAT category), the cardiopulmonary bypass (CPB) times, and the cross-clamp times, were collected from the STS database and EHR.

### 2.3. Study Design

**Figure 1:**
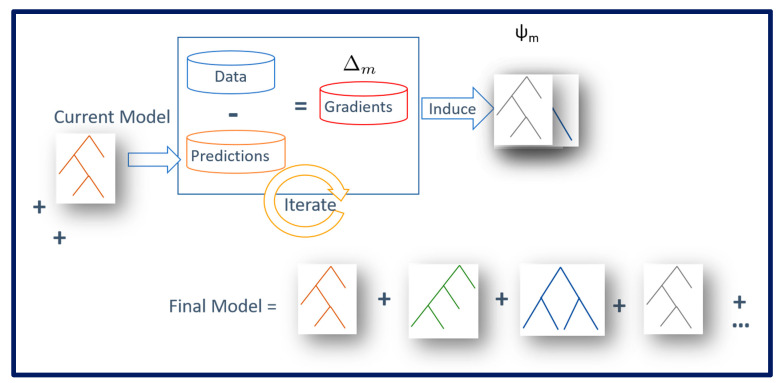
Illustration of functional gradient boosting where the classifier is constructed in a stage-wise process. At each iteration, prediction errors are computed for each training example based on the current model. Then, a simple regression model (typically a small tree) is created to correct the errors and the process is repeated until convergence. In contrast to standard gradient-based methods, the functional gradients are point-wise gradients computed for each example separately as against the entire data set. This allows for both efficient and effective learning from large data sets.

To learn a model that predicts cardiac arrest in children in the CICU, using a relatively few patient examples, we used the RDN Boost framework [14], which is a non-parametric ensemble machine learning algorithm. In this framework, regression tree weak learners were created, aiming to learn the probability that a patient example will suffer from an IHCA. As depicted in Figure 1, the current model (consisting of the trees learned thus far) was used to compute the prediction error (formally the gradient), and new trees were computed that reduced the predictive error. The final model was the sum of the regression trees. Each example would only satisfy one path of the tree, and the corresponding leaf value was added to the overall regression value at each tree. This is to say that each example would get a regression value from each tree, and the final conditional probability was proportional to the sum of the regression values from all the trees. 

Such ensemble methods demonstrate a high robustness, especially in settings where there are not enough well-balanced, high-quality, labelled data to train more data-intensive models, such as deep neural networks (DNNs). This is due to the fact that the ensemble methods can discover weak associations between the risk factors in constructing a strong association model. The combination of these weak factors can result in possibly stronger hypotheses, specifically in the context of a smaller number of data samples. One advantage of the RDN framework, which is commonly used for the inductive learning of models over the data from a relational database such as an EHR (electronic health record), is the robustness of the missing data. Unlike the setting where the data must be incorporated into vectors for the downstream algorithms, here, we encode data in the form of predicates; for example, the predicate pH (subj1, T, 7.4) states that, for the particular patient subj1, the pH is 7.4 at time T. A potential disadvantage of this encoding is the necessary discretization of the real-valued data into pre-determined bins. This may not impact the results of this clinical exercise, in that physicians are used to dealing with normal ranges of laboratory data; for example, a pH of 7.40 would not generally be regarded as different from a value of 7.41. However, depending on the size of the ‘buckets’ into which the values are discretized, we may not be able to discover the small, but clinically important, trends in the data.

We initially used the XGBoost [15] algorithm to ascertain which features were the most important to a standard computer prediction model. While one could imagine using other feature selection strategies, including the computing of mutual information, given that we used a relational gradient boosting in the second step, using the feature based gradient boosting to simply reduce the number of features was a natural choice. Moreover, XGBoost uses a set of criteria to identify the features to split on (including percentiles), and thus serves as a natural feature selection strategy compared to picking a specific method. The top eleven selected features were: heart rate (HR), VIS, oxygen saturation (SpO_2_), urine output (in mL/kg/hour) diastolic blood pressure (DBP), end tidal carbon dioxide (ETCO_2_), cerebral oximetry (rSO_2_c), somatic oximetry (rSO_2_s), anion gap, base excess, and fractional inspired oxygen (FiO_2_). The 11 variables, in order from the most to least missing data, were the following: anion gap, FiO_2_, base excess, ETCO_2_, rSO_2_s, rSO_1_c, DBP, SpO_2_, HR, urine output, and VIS. The following features were normalized (change from baseline) to the first four hours of the data that had been collected for each patient: HR, DBP, SpO_2_, rSO_2_c, and rSO_2_s. The reason for the HR and DBP normalization was because the HR and DBP could vary based on age. The reason for the SpO_2_, rSO_2_c, and rSO_2_s normalization was because these vital signs could vary based on whether the patient had cyanotic or noncyanotic heart disease and the degree of the right to left shunt. 

RDN Boost is the relational extension of XGBoost (i.e., it extends the standard gradient boosting to handle relational data). So, using XGBoost, we extracted the most informative features and then constructed the predicate format to allow for a richer representation. Using the RDN Boost algorithm, we created a set of regression trees, whose cardinality grew as more clinical data accumulated in the EHR. The data were collected in increments of several hours, and at each increment, new trees were concatenated to the previous model in a stacking fashion, improving the predictive ability with time. For all the features, we discretized the continuous variables into three bins.

The fact that we created distinct sets of trees for different time periods is an important feature of our model. Owing to this design, we can use the same model (limiting the number of trees as needed) for patients in whom only a few hours of data have been accumulated. This is in contrast to vector-based classification models (such as XG-Boost), which require the creation of different models as the length of the feature vectors is altered.

The goal of the model was to predict the probability of an IHCA over the 13 h prior to a CA. We trained the model using data from the 16 h prior to the IHCA in the patients who arrested and the last 16 h of data in the controls. For each 4 h of data, we created a set of five boosted regression trees. For each subsequent four-hour period, we created another five trees, using all the data from the hours before. We performed five replicants of the experiments, subsampling the non-arrest group to account for the class imbalance in the data. 

This analysis is particularly important, as it demonstrates the “anytime” capabilities of our boosting algorithm. Specifically, the algorithm provides the ability to query the model any time before an event occurs, where there are at least 4 h of data. Given the set of observations leading up to the time of querying, the algorithm gives the “best” estimate of the risk of cardiac arrest. As the observations increase, i.e., as more time passes, the estimate becomes closer to the true probability of the arrest. The algorithm is anytime, i.e., it can be easily proved that the “estimate” only improves with time and more observations. This is crucial in building the trust with clinicians as the prediction improves with time. Another salient feature of the model is that it can handle missing data, even at the query time, and can still yield robust predictions. 

We also compared the results of our functional gradient boosting algorithm to that of a Gaussian Naïve Bayes classifier, where we used the numerical values of the features without discretization; the missing data values were imputed with the most recent recorded value in a ‘forward imputation’ strategy.

## 3. Results

**Figure 2:**
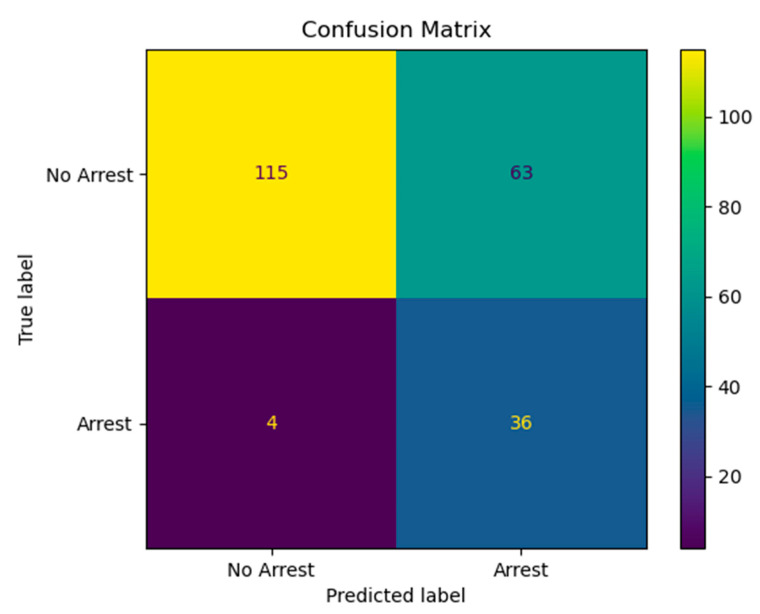
Confusion matrix: the model had a sensitivity of 90% and a specificity of 65%. The model had a positive predictive value of 36% and a negative predictive value of 97%.

There were 160 index events during the study period. We selected 711 unique control patients who met the inclusion criteria. Our group had previously published the demographics of those patients who had an IHCA vs. no IHCA [4]. The patients who had an IHCA tended to be younger, lower in weight, more likely to have single-ventricle physiology, and had undergone higher STAT category cardiac surgical procedures, with longer CPB and cross-clamp times. Figure 2 shows a representative confusion matrix from a representative fold of the predictive model. 

**Figure 3:**
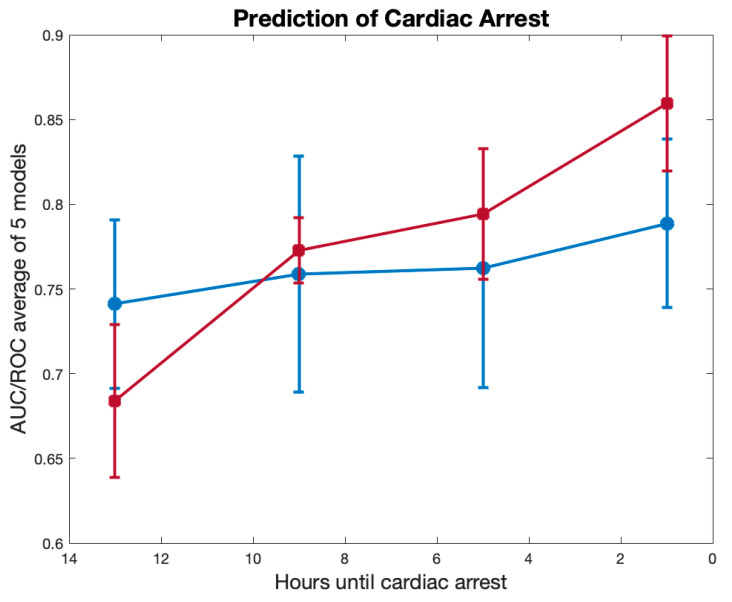
Comparison of the predictive machine learning algorithm (red) to the Gaussian Naïve Bayes model (blue). The x axis represents the number of hours until the cardiac arrest. The y axis represents the AUROC of the corresponding model at the corresponding hour.

We compared the results of these experiments to those that were obtained using the Gaussian Naïve Bayes classifier. The results of these experiments are presented in Figure 3, demonstrating a considerable performance enhancement in the non-parametric boosting model using the predicate data, when compared to the parametric naïve Bayes predictive model.

Figure 3 shows the concatenated model with the AUROC as a function of the hours until the IHCA, with a standard deviation that was generated using a 5-fold cross validation. 

The red line shows the boosted concatenated model and the blue line shows the Gaussian Naïve Bayes model. As expected, as the time gets closer to the CA, the prediction of the model becomes better. At one hour prior to the CA, the AUROC was the highest, at 0.85 [0.79,0.90]. 

### 3.1. Feature Importance

We attempted to ascertain which of the features contributed most strongly to the model prediction. This is challenging in models consisting of weak learners that cooperate in an ensemble fashion. Such models are very good at synthesizing multiple features, and the contribution of single features can be quite low. Inspired by the recent work of Lundberg and Lee [16], we grouped the eleven parameters into different combinations. We then tested the model’s performance using only the features of each group. Table 1 shows the AUROC of the model for hours 16-13, 16-9, 16-5, and 16-1, encoded with our stacked models of 5, 10, 15, and 20 trees. The standard deviations were generated using 5-fold validation. 

It can be easily observed that the contribution of the feature group that consisted of SpO_2_, ETCO_2_, FiO_2_, anion gap, and base excess had the worst performance, and the group consisting of HR, DBP, rSO_2_c, and VIS performed the best (nearly as good as the model with all 11 features) at 1 h prior to arrest. We then removed the individual features from each grouping to try to delineate the importance of each. We noted that the removal of the VIS from the best-performing subgroup resulted in a decline in the prediction, which is not surprising, since the use of vasoactive medications can affect the other variables in its group. Another interesting finding was that a feature group that already had the variable rSO_2_c did not perform any better with the addition of the variable rSO_2_s. Overall, the parameters measuring the indices of perfusion were most important, and resulted in the best performance in our boosted predictive model.

### 3.2. Logistic Regression Model

We compared our predictive algorithm to a multivariable logistic regression model. The results of the performance of the logistic regression model have been previously published [4]. We used the same eleven features that were found to be most important to the initial XG Boost algorithm, and used a univariate analysis on the ability to predict cardiac arrest at each hour, for the preceding 15 h prior to the cardiac arrest. If there were multiple values within an hour, we used the mean of the values within that hour. The missing values were imputed forward in time from the last documented value. Again, just as in our predictive algorithm, we normalized the HR, DBP, SpO_2_, rSO_2_c, and rSO_2_s to the first 4 h of the data (considered to be the baseline). The values for the VIS were not normally distributed and therefore it could not be represented as a continuous variable. The values for the VIS were skewed, with a median VIS of 0, therefore, we elected to classify the values for the VIS into two categories, VIS 0 and VIS > 0. We created a multivariate model using the DBP, HR, VIS, and SpO_2_-rSO_2_c difference, and the SpO_2_-rSO_2_s difference. Our multivariate model had an AUC of 0.83 one hour prior to CA. As in our boosted model, the predictive ability improved with the acquisition of data closer to the time of cardiac arrest.

## 4. Discussion

Using single-center data, we created a non-parametric machine learning algorithm that could predict an IHCA within 1 h, using only 11 variables that were derived from the EHR, with a AUROC of 0.85 [0.79,0.90]. A unique aspect of our algorithm is that the model was altered as each tranche of data arrived, improving its predictive accuracy and decreasing the model variance. This was owing to the boosted nature of the concatenating weakly performing classifiers. Unlike typical predictive models, where the features are collected over a particular time period, our model used recent data over previous data to add to the model, thus making it able to remember important features and events from earlier times in the clinical trajectory, improving its performance over time. It is not surprising that our machine learning algorithm performed similarly to the logistic regression model, given that both models used the same 11 variables that were identified by the XG Boost algorithm to be most important for the IHCA prediction. 

The most important features for the predictive algorithm were the HR, DBP, SpO_2_, rSO_2_c, and VIS. In fact, this group performed almost as well as the model with all 11 variables. Although our predictive algorithm will not explain why a patient is at a high risk of an IHCA, it will alert a clinician to pay more attention to those key features on a decompensating patient, and allow for earlier investigation and a potential intervention to avert an IHCA. The only two features of our model that involved medical intervention rather than changes in the patient themselves were FiO_2_ and VIS. It was not surprising that the VIS was found to be an important feature, since an elevated VIS has been shown in prior studies to be associated with worse outcomes in children after cardiac surgery [17]. The addition of the VIS to the group with the SpO_2_, HR, DBP, rSO_2_s, and rSO_2_c improved its performance, since a clinician was actively intervening with hypotension. Another interesting finding was that the removal of the variable rSO_2_s from a group that already had the variable rSO_2_c made no difference in its performance. Typically, when the cardiac output is limited, as it is in the prearrest state, the body’s response is to limit somatic perfusion in order to preserve cerebral perfusion [18]. Thus, we would anticipate the changes in rSO_2_s to happen earlier than the changes in rSO_2_c, which may only occur minutes prior to an IHCA, and may not necessarily be seen in our model using hourly vital signs. A possible explanation of the relative importance of rSO_2_c compared to rSO_2_s in our machine learning model may be related to the inherent differences in the cardiac arrest vs. control groups. As noted, the arrest patients tended to be younger, more likely to have single-ventricle physiology, and if they were post-surgical, had higher STAT categories, which are all known risk factors for impaired cerebral autoregulation [19,20]. Thus, our predictive model may have detected changes in rSO_2_c which were the result of impaired cerebral autoregulation, rather than a sign of an impending IHCA. It is difficult to ascertain the cause of this, since impaired cerebral autoregulation can also occur in an impending IHCA as well. 

Given the low prevalence of IHCAs in our unit (the rate of IHCA in our CICU is 3 events per 1000 patient days, which is approximately 20 IHCA events per year), our predictive algorithm will understandably have a low positive predictive value (PPV) and a high negative predictive value (NPV). Even though our predictive algorithm had a low PPV, the high NPV can be helpful as a bedside tool, especially in times of decreased clinician presence, such as nights and weekends. Our work has also shown that machine learning algorithms can be successfully applied to small datasets with a class imbalance, which is a common problem due to case vs. control balancing when the number of events (cardiac arrest) is an order of magnitude smaller (as in pediatric congenital heart care). 

Our study is the largest study to date to evaluate the use of machine learning algorithms in predicting the cardiac arrests in a pediatric CICU population. Our study included 160 patients with index cardiac arrest events and 711 control patients with approximately 41,000 h of data. While other machine learning studies on pediatric CICU populations evaluated more homogenous patients (single-ventricle physiology or infants < 1 year of age), ours was a heterogenous population that was made up of a wide variety, which can be seen in a typical large academic pediatric CICU. We included patients with single-ventricle and non-single-ventricle physiology, all their ages were <18 years of age, and postsurgical and medical CICU admissions were also used. While we would have liked to perform subgroup analyses to evaluate if the performance of our machine learning algorithm was improved in certain groups, such as medical vs. post-surgical admissions, different etiologies of IHCA (respiratory vs. non-respiratory), or high-risk cardiac diagnoses such as single-ventricle physiology, unfortunately, the small numbers in our cohort prevented us from performing these further analyses. This will be our goal in future analyses as we collect more patient data. 

### Limitations

One of the limitations of our study is that it was a single-center retrospective study. Another limitation is that our predictive algorithm did not use continuous physiologic data, but rather hourly data that were retrieved by the EHR which is prone to inaccuracies given that it is dependent on the manual verification of the data that are pulled from the central monitor into the EHR. Despite this limitation, we believe this can be also seen as a strength, since all the data were derived from the EHR, meaning that other important variables, such as medications, could be incorporated into our predictive algorithm and programmed into the EHR, which can alert clinicians of an impending IHCA.

## 5. Conclusions

With the use of the 11 vital sign and laboratory variables that were collected from the EHR, with historical training/testing sets, we successfully created a machine learning predictive algorithm to predict the IHCAs in children that had been admitted to our single-center pediatric CICU, with a AUROC of 0.85 [0.79,0.90] one hour prior to the CA. Our predictive algorithm’s performance was comparable to a traditional logistic regression model. The results of this analysis provide a solid foundation for the development of a machine learning algorithm to predict IHCAs in children with CHD, which could be applied at the point of care. Future research needs a validation of the model, with the addition of high-frequency physiologic data, as well as prospectively collected and multicenter data, prior to its implementation at the bedside.

## Figures and Tables

**Table 1:** Model performance by subgroups of features.

Groups	AUROC for Hours			
	13	9	5	1
SpO_2_, ETCO_2_, anion gap, base excess, FiO_2_	0.63 ± 0.04	0.69 ± 0.03	0.75 ± 0.03	0.81 ± 0.02
HR, DBP, SpO_2_ rSO_2_c	0.67 ± 0.05	0.73 ± 0.06	0.80 ± 0.03	0.84 ± 0.03
HR, DBP, SpO_2_ rSO_2_s	0.68 ± 0.02	0.71 ± 0.01	0.78 ± 0.03	0.81 ± 0.02
HR, DBP, SpO_2_, rSO_2_c, rSO_2_s	0.69 ± 0.02	0.69 ± 0.03	0.79 ± 0.02	0.85 ± 0.02
HR, DBP, SpO_2_, rSO_2_c, rSO_2_s, VIS	0.69 ± 0.05	0.72 ± 0.03	0.80 ± 0.03	0.87 ± 0.02
HR, DBP, SpO_2_, rSO_2_c, VIS	0.66 ± 0.03	0.72 ± 0.02	0.78 ± 0.01	0.87 ± 0.03
HR, DBP, VIS, urine output	0.71 ± 0.02	0.73 ± 0.04	0.79 ± 0.03	0.84 ± 0.01

## Data Availability

The data presented in this study are available on request from the corresponding author.

## Supplementary Materials

The following supporting information can be downloaded at: https://www.mdpi.com/article/10.3390/jcm12072728/s1, Table S1: Potential Study Features and Frequency Collected from Electronic Health Record.

## References

[1] Lowry A.W., Knudson J.D., Cabrera A.G., Graves D.E., Morales D.L., Rossano J.W. (2013). Cardiopulmonary resuscitation in hospitalized children with cardiovascular disease: Estimated prevalence and outcomes from the kids’ inpatient database. Pediatr. Crit. Care Med..

[2] Alten J.A., Klugman D., Raymond T.T., Cooper D.S., Donohue J.E., Zhang W., Pasquali S.K., Gaies M.G. (2017). Epidemiology and Outcomes of Cardiac Arrest in Pediatric Cardiac ICUs. Pediatr. Crit. Care Med..

[3] Marino B.S., Tabbutt S., MacLaren G., Hazinski M.F., Adatia I., Atkins D.L., Checchia P.A., DeCaen A., Fink E.L., Hoffman G.M. (2018). Cardiopulmonary Resuscitation in Infants and Children with Cardiac Disease: A Scientific Statement From the American Heart Association. Circulation.

[4] Yu P., Esangbedo I., Li X., Wolovits J., Thiagarajan R., Raman L. (2022). Early Changes in Near-Infrared Spectroscopy Are Associated with Cardiac Arrest in Children with Congenital Heart Disease. Front. Pediatr..

[5] Layeghian Javan S., Sepehri M.M., Aghajani H. (2018). Toward analyzing and synthesizing previous research in early prediction of cardiac arrest using machine learning based on a multi-layered integrative framework. J. Biomed. Inform..

[6] Matam B.R., Duncan H., Lowe D. (2019). Machine learning based framework to predict cardiac arrests in a paediatric intensive care unit: Prediction of cardiac arrests. J. Clin. Monit. Comput..

[7] Kennedy C.E., Aoki N., Mariscalco M., Turley J.P. (2015). Using Time Series Analysis to Predict Cardiac Arrest in a PICU. Pediatr. Crit. Care Med..

[8] Rusin C.G., Acosta S.I., Shekerdemian L.S., Vu E.L., Bavare A.C., Myers R.B., Patterson L.W., Brady K.M., Penny D.J. (2016). Prediction of imminent, severe deterioration of children with parallel circulations using real-time processing of physiologic data. J. Thorac. Cardiovasc. Surg..

[9] Ruiz V.M., Saenz L., Lopez-Magallon A., Shields A., Ogoe H.A., Suresh S., Munoz R., Tsui F.R. (2019). Early prediction of critical events for infants with single-ventricle physiology in critical care using routinely collected data. J. Thorac. Cardiovasc. Surg..

[10] Bose S.N., Verigan A., Hanson J., Ahumada L.M., Ghazarian S.R., Goldenberg N.A., Stock A., Jacobs J.P. (2019). Early identification of impending cardiac arrest in neonates and infants in the cardiovascular ICU: A statistical modelling approach using physiologic monitoring data-CORRIGENDUM. Cardiol. Young.

[11] Ruiz V.M., Goldsmith M., Shi L., Simpao A., Galvaz J., Naim M., Nadkarni V., Gaynor W., Tsui F.R. (2021). Early prediction of clinical deterioration using data-driven machine learning modeling of electronic health records. J. Thorac. Cardiovasc. Surg..

[12] Raedt L.D., Kersting K., Natarajan S., Poole D. (2016). Statistical relational artificial intelligence: Logic, probability, and computation. Synth. Lect. Artif. Intell. Mach. Learn..

[13] Schwartz S.M., Dent C.L., Musa N.L., Nelson D.P. (2003). Single-ventricle physiology. Crit. Care Clin..

[14] Natarajan S., Khot T., Kersting K., Gutmann B., Shavlik J. (2012). Gradient-based boosting for statistical relational learning: The relational dependency network case. Mach. Learn..

[15] Chen T., Guestrin C. XGBoost: A Scalable Tree Boosting System. Proceedings of the 22nd ACM SIGKDD International Conference on Knowledge Discovery and Data Mining.

[16] Lundberg S.M., Lee S.I. (2017). A unified approach to interpreting model predictions. Adv. Neural Inf. Process. Syst..

[17] Gaies M.G., Jeffries H.E., Niebler R.A., Pasquali S.K., Donohue J.E., Yu S., Gall C., Rice T.B., Thiagarajan R.R. (2014). Vasoactive-inotropic score is associated with outcome after infant cardiac surgery: An analysis from the Pediatric Cardiac Critical Care Consortium and Virtual PICU System Registries. Pediatr. Crit. Care Med..

[18] Bonanno F.G. (2011). Clinical pathology of the shock syndromes. J. Emerg. Trauma Shock.

[19] Spaeder M.C., Klugman D., Skurow-Todd K., Glass P., Jonas R.A., Donofrio M.T. (2017). Perioperative Near-Infrared Spectroscopy Monitoring in Neonates with Congenital Heart Disease: Relationship of Cerebral Tissue Oxygenation Index Variability with Neurodevelopmental Outcome. Pediatr. Crit. Care Med..

[20] Spilka J.M., O’Halloran C.P., Marino B.S., Brady K.M. (2021). Perspective on Cerebral Autoregulation Monitoring in Neonatal Cardiac Surgery Requiring Cardiopulmonary Bypass. Front. Neurol..

