# Supplementary material for: Predicting Cardiac Arrest in Children with Heart Disease: A Novel Machine Learning Algorithm
Source: J Clin Med. 2023 Apr 6;12(7):2728. doi: 10.3390/jcm12072728 (PMC10095110; doi:10.3390/jcm12072728)
Supplement: Supplementary file 1 [file jcm-12-02728-s001.zip › jcm-2233754-supplementary.pdf]

Supplemental Table S1: Potential Study Features and Frequency Collected from Electronic Health Record

Vital Signs	Laboratory data	medications	Ventilator settings	Echocardiogram parameters	Other
Systolic blood pressure (hourly)	Blood gas (variable)	Vasoactive infusions (hourly)	Fraction of inspired oxygen (hourly)	Systemic ventricle function (variable)	Urine output (variable)
Mean arterial blood pressure (hourly)	Basic metabolic panel (variable)	Sedative/ neuromuscular blockade infusions (hourly)	Peak inspiratory pressure (hourly)	Systemic ventricle atrioventricular valve regurgitation (variable)	
Diastolic blood pressure (hourly)	Liver function panel (variable)	Diuretics (variable)	Positive end expiratory pressure (hourly)	Subpulmonary ventricle function (variable)	
Heart rate (hourly)	Coagulation studies (variable)	Antiarrhythmic infusions (hourly)	Mean airway pressure (hourly)	Subpulmonary ventricle hypertension (variable)	
Central venous/intracardiac pressure (hourly)	Complete blood count (variable)		Tidal volume (hourly)	Tricuspid regurgitation (variable)	
End tidal carbon dioxide level (hourly)				Pulmonary vein obstruction (variable)	
Oxygen saturation (hourly)				Aortic insufficiency (variable)	
Cerebral oximetry (hourly)					
Somatic oximetry (hourly)					

Minimum frequency of variable collected in parenthesis
